# 1,4-Bis{[5-(pyridin-4-yl)-1,3,4-oxadiazol-2-yl]sulfan­yl}butane

**DOI:** 10.1107/S1600536811006805

**Published:** 2011-02-26

**Authors:** Qing-lei Liu, Wei Wang, Yan Gao, Xiao-yu Jia, Jing-jing Zhang

**Affiliations:** a School of Perfume and Aroma Technology, Shanghai Istitute of Technology, Shanghai 200235, People’s Republic of China; b School of Chemical Engineering, University of Science and Technology LiaoNing, Anshan 114051, People’s Republic of China

## Abstract

In the centrosymmetric title compound, C_18_H_16_N_6_O_2_S_2_, the 1,3,4-oxadiazole and the attached pyridinyl ring are twisted by 5.3 (3)°.

## Related literature

For applications of heterocyclic derivatives, see: Al-Talib *et al.* (1990 ▶); Nakagawa *et al.* (1996 ▶); Zhang *et al.* (2007 ▶). For related structuresbn,, see: Wang *et al.* (2010 ▶, 2011 ▶); Zhao *et al.* (2010 ▶).
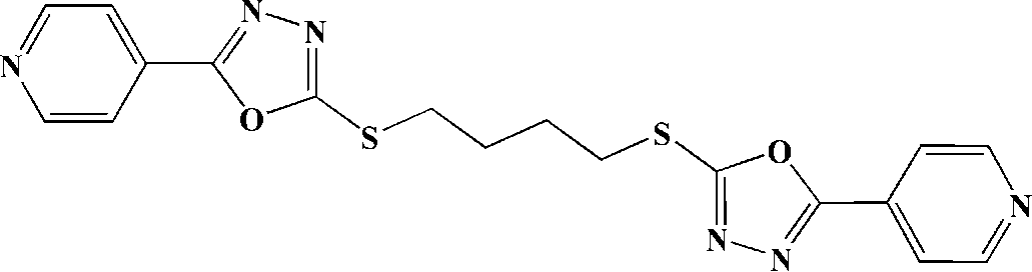


## Experimental

### 

#### Crystal data

C_18_H_16_N_6_O_2_S_2_
*M* *_r_* = 412.49
Monoclinic, 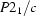

*a* = 4.9780 (6) Å
*b* = 5.7933 (7) Å
*c* = 31.003 (4) Å
β = 92.588 (5)°
*V* = 893.20 (18) Å^3^
*Z* = 2
Mo *K*α radiation
μ = 0.33 mm^−1^
*T* = 113 K
0.20 × 0.18 × 0.10 mm

#### Data collection

Rigaku Saturn CCD area-detector diffractometer
Absorption correction: multi-scan (*CrystalClear*; Rigaku/MSC, 2005 ▶) *T*_min_ = 0.937, *T*_max_ = 0.962
8437 measured reflections
2128 independent reflections
1811 reflections with *I* > 2σ(*I*)
*R*_int_ = 0.035

#### Refinement

*R*[*F* ^2^ > 2σ(*F* ^2^)] = 0.032
*wR*(*F* ^2^) = 0.089
*S* = 1.10
2128 reflections
127 parameters
H-atom parameters constrained
Δρ_max_ = 0.40 e Å^−3^
Δρ_min_ = −0.19 e Å^−3^

### 

Data collection: *CrystalClear* (Rigaku/MSC, 2005 ▶); cell refinement: *CrystalClear*; data reduction: *CrystalClear*; program(s) used to solve structure: *SHELXS97* (Sheldrick, 2008 ▶); program(s) used to refine structure: *SHELXL97* (Sheldrick, 2008 ▶); molecular graphics: *SHELXTL* (Sheldrick, 2008 ▶); software used to prepare material for publication: *SHELXTL*.

## Supplementary Material

Crystal structure: contains datablocks global, I. DOI: 10.1107/S1600536811006805/kp2310sup1.cif

Structure factors: contains datablocks I. DOI: 10.1107/S1600536811006805/kp2310Isup2.hkl

Additional supplementary materials:  crystallographic information; 3D view; checkCIF report

## Figures and Tables

**Fig. 1.**
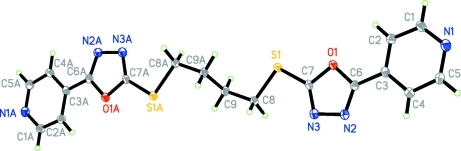
View of the molecule of (I) showing the atom-labelling scheme [symmetry code: (A)-x, -y + 1, -z + 1]. Displacement ellipsoids are drawn at the 50% probability level.

## supplementary crystallographic information

### Comment

Heterocycle derivatives containing N, O and S atoms are under intensive studies due to their wide applications in medicine, industry and coordination chemistry (Al-Talib *et al.*, 1990; Nakagawa *et al.*, 1996; Zhang *et al.*, 2007). We are focusing the synthetic and structural studies on the novel thio-based ligands (Wang *et al.*, 2010, 2011; Zhao *et al.*, 2010). Here we present the synthesis and the crystal structure of the title compound (I), namely, 1,4-bis[5-(pyridin-4-yl)-1,3,4-oxadiazol-2-ylsulfanyl]butane.

The molecular structure of title compound (I) (Fig. 1)reveals a twofold rotational axis through the mid of the C-C bond of butane group. Therefore, an asymmetric unit comprises a half of the molecule. 1,3,4-Oxadiazole moiety is planar with an r.m.s. deviation of 0.0033 (2)Å and maximum deviation of 0.0052 (2)Å for the atom C7. The dihedral angle between the oxadiazole and its attached pyridinyl ring [r.m.s. deviation = 0.0062 (2) Å] of 5.3 (3)° indicates that they are almost coplanar. As a result of π-π conjugation, the C*_sp_*^2^-S bond [S1—C7 = 1.722 (13) Å] is significantly shorter than the C*_sp_*^3^-S bond [S1—C8 = 1.817 (12) Å].

### Experimental

A suspension of 5-(pyridin-4-yl)-1,3,4-oxadiazole-2-thiol (2.0 mmol) and 1,1-dibromobutane (1.0 mmol) in ethanol (10 mL) was stirred at room temperature. The reaction progress was monitored *via* TLC. The resulting precipitate was filtered off, washed with cold ethanol, dried and purified to give the target product as light-yellow solid in 87% yield. Crystals of (I) suitable for single-crystal X-ray analysis were grown by slow evaporation of a solution in chloroform-ethanol (1:1).

### Refinement

All H atoms were positioned geometrically and refined as riding (C—H = 0.95–0.99 Å) and allowed to ride on their parent atoms, with *U*_iso_(H) = 1.2*U*_eq_(parent).

### Crystal data

**Table:**

C_18_H_16_N_6_O_2_S_2_	*F*(000) = 428
*M**_r_* = 412.49	*D*_x_ = 1.534 Mg m^−^^3^
Monoclinic, *P*2_1_/*c*	Mo *K*α radiation, λ = 0.71073 Å
Hall symbol: -P 2ybc	Cell parameters from 2798 reflections
*a* = 4.9780 (6) Å	θ = 2.6–27.9°
*b* = 5.7933 (7) Å	µ = 0.33 mm^−^^1^
*c* = 31.003 (4) Å	*T* = 113 K
β = 92.588 (5)°	Prism, light-yellow
*V* = 893.20 (18) Å^3^	0.20 × 0.18 × 0.10 mm
*Z* = 2	

### Data collection

**Table:**

Rigaku Saturn CCD area-detector diffractometer	2128 independent reflections
Radiation source: rotating anode	1811 reflections with *I* > 2σ(*I*)
multilayer	*R*_int_ = 0.035
Detector resolution: 14.63 pixels mm^-1^	θ_max_ = 27.9°, θ_min_ = 2.6°
φ and ω scans	*h* = −4→6
Absorption correction: multi-scan (*CrystalClear*; Rigaku/MSC, 2005)	*k* = −7→7
*T*_min_ = 0.937, *T*_max_ = 0.962	*l* = −38→40
8437 measured reflections	

### Refinement

**Table:**

Refinement on *F*^2^	Primary atom site location: structure-invariant direct methods
Least-squares matrix: full	Secondary atom site location: difference Fourier map
*R*[*F*^2^ > 2σ(*F*^2^)] = 0.032	Hydrogen site location: inferred from neighbouring sites
*wR*(*F*^2^) = 0.089	H-atom parameters constrained
*S* = 1.10	*w* = 1/[σ^2^(*F*_o_^2^) + (0.0531*P*)^2^ + 0.054*P*] where *P* = (*F*_o_^2^ + 2*F*_c_^2^)/3
2128 reflections	(Δ/σ)_max_ = 0.001
127 parameters	Δρ_max_ = 0.40 e Å^−^^3^
0 restraints	Δρ_min_ = −0.19 e Å^−^^3^

### Special details

**Table:**

Geometry. All e.s.d.'s (except the e.s.d. in the dihedral angle between two l.s. planes) are estimated using the full covariance matrix. The cell e.s.d.'s are taken into account individually in the estimation of e.s.d.'s in distances, angles and torsion angles; correlations between e.s.d.'s in cell parameters are only used when they are defined by crystal symmetry. An approximate (isotropic) treatment of cell e.s.d.'s is used for estimating e.s.d.'s involving l.s. planes.
Refinement. Refinement of *F*^2^ against ALL reflections. The weighted *R*-factor *wR* and goodness of fit *S* are based on *F*^2^, conventional *R*-factors *R* are based on *F*, with *F* set to zero for negative *F*^2^. The threshold expression of *F*^2^ > σ(*F*^2^) is used only for calculating *R*-factors(gt) *etc*. and is not relevant to the choice of reflections for refinement. *R*-factors based on *F*^2^ are statistically about twice as large as those based on *F*, and *R*- factors based on ALL data will be even larger.

### Fractional atomic coordinates and isotropic or equivalent isotropic displacement parameters (Å^2^)

**Table:**

	*x*	*y*	*z*	*U*_iso_*/*U*_eq_	
S1	0.25227 (6)	0.35176 (5)	0.422550 (9)	0.01776 (12)	
O1	0.61211 (17)	0.17771 (15)	0.37253 (3)	0.0163 (2)	
N1	1.3455 (2)	−0.0526 (2)	0.27364 (3)	0.0197 (2)	
N2	0.7346 (2)	−0.15583 (19)	0.40229 (3)	0.0186 (2)	
N3	0.5348 (2)	−0.05632 (19)	0.42735 (3)	0.0189 (2)	
C1	1.1858 (3)	0.1307 (2)	0.27715 (4)	0.0190 (3)	
H1	1.2063	0.2551	0.2576	0.023*	
C2	0.9910 (3)	0.1507 (2)	0.30774 (4)	0.0179 (3)	
H2	0.8797	0.2836	0.3086	0.021*	
C3	0.9634 (2)	−0.0287 (2)	0.33688 (4)	0.0155 (3)	
C4	1.1268 (3)	−0.2220 (2)	0.33375 (4)	0.0185 (3)	
H4	1.1125	−0.3479	0.3531	0.022*	
C5	1.3118 (3)	−0.2262 (2)	0.30146 (4)	0.0199 (3)	
H5	1.4206	−0.3598	0.2990	0.024*	
C6	0.7718 (2)	−0.0138 (2)	0.37112 (4)	0.0153 (3)	
C7	0.4729 (2)	0.1379 (2)	0.40882 (4)	0.0152 (3)	
C8	0.1260 (3)	0.2236 (2)	0.47114 (4)	0.0177 (3)	
H8A	0.0331	0.0764	0.4640	0.021*	
H8B	0.2770	0.1908	0.4921	0.021*	
C9	−0.0695 (3)	0.3927 (2)	0.49063 (4)	0.0178 (3)	
H9A	−0.2052	0.4403	0.4680	0.021*	
H9B	−0.1652	0.3137	0.5137	0.021*	

### Atomic displacement parameters (Å^2^)

**Table:**

	*U*^11^	*U*^22^	*U*^33^	*U*^12^	*U*^13^	*U*^23^
S1	0.0216 (2)	0.01715 (19)	0.01492 (18)	0.00366 (11)	0.00549 (12)	0.00101 (11)
O1	0.0174 (4)	0.0179 (5)	0.0140 (4)	0.0017 (3)	0.0045 (3)	0.0003 (3)
N1	0.0180 (5)	0.0223 (6)	0.0191 (5)	−0.0024 (4)	0.0030 (4)	−0.0030 (4)
N2	0.0208 (6)	0.0179 (6)	0.0176 (5)	0.0020 (4)	0.0053 (4)	−0.0002 (4)
N3	0.0201 (5)	0.0193 (6)	0.0177 (5)	0.0022 (4)	0.0053 (4)	−0.0003 (4)
C1	0.0194 (7)	0.0202 (7)	0.0176 (6)	−0.0024 (5)	0.0037 (5)	0.0011 (5)
C2	0.0178 (6)	0.0173 (6)	0.0186 (6)	0.0012 (5)	0.0023 (5)	−0.0005 (5)
C3	0.0146 (6)	0.0185 (6)	0.0132 (5)	−0.0014 (5)	−0.0001 (4)	−0.0016 (4)
C4	0.0205 (6)	0.0185 (6)	0.0166 (6)	0.0009 (5)	0.0022 (5)	0.0009 (5)
C5	0.0193 (6)	0.0200 (6)	0.0207 (6)	0.0025 (5)	0.0029 (5)	−0.0026 (5)
C6	0.0142 (6)	0.0160 (6)	0.0158 (6)	0.0011 (4)	0.0003 (4)	−0.0017 (4)
C7	0.0155 (6)	0.0188 (6)	0.0114 (5)	−0.0013 (4)	0.0028 (4)	−0.0014 (4)
C8	0.0215 (6)	0.0162 (6)	0.0158 (6)	−0.0010 (5)	0.0055 (5)	−0.0007 (5)
C9	0.0173 (6)	0.0182 (6)	0.0183 (6)	−0.0013 (5)	0.0042 (5)	−0.0030 (5)

### Geometric parameters (Å, °)

**Table:**

S1—C7	1.7218 (13)	C2—H2	0.9500
S1—C8	1.8171 (12)	C3—C4	1.3902 (17)
O1—C6	1.3669 (14)	C3—C6	1.4615 (16)
O1—C7	1.3675 (14)	C4—C5	1.3906 (17)
N1—C1	1.3337 (17)	C4—H4	0.9500
N1—C5	1.3404 (17)	C5—H5	0.9500
N2—C6	1.2888 (16)	C8—C9	1.5251 (17)
N2—N3	1.4121 (15)	C8—H8A	0.9900
N3—C7	1.2941 (16)	C8—H8B	0.9900
C1—C2	1.3914 (18)	C9—C9^i^	1.525 (2)
C1—H1	0.9500	C9—H9A	0.9900
C2—C3	1.3878 (17)	C9—H9B	0.9900
			
C7—S1—C8	99.16 (6)	C4—C5—H5	118.1
C6—O1—C7	101.91 (9)	N2—C6—O1	113.00 (10)
C1—N1—C5	116.91 (11)	N2—C6—C3	128.89 (11)
C6—N2—N3	106.29 (10)	O1—C6—C3	118.07 (10)
C7—N3—N2	105.69 (10)	N3—C7—O1	113.10 (11)
N1—C1—C2	123.91 (12)	N3—C7—S1	131.13 (10)
N1—C1—H1	118.0	O1—C7—S1	115.76 (9)
C2—C1—H1	118.0	C9—C8—S1	108.47 (9)
C3—C2—C1	118.29 (12)	C9—C8—H8A	110.0
C3—C2—H2	120.9	S1—C8—H8A	110.0
C1—C2—H2	120.9	C9—C8—H8B	110.0
C2—C3—C4	118.84 (11)	S1—C8—H8B	110.0
C2—C3—C6	121.06 (11)	H8A—C8—H8B	108.4
C4—C3—C6	120.08 (11)	C9^i^—C9—C8	112.82 (13)
C3—C4—C5	118.21 (12)	C9^i^—C9—H9A	109.0
C3—C4—H4	120.9	C8—C9—H9A	109.0
C5—C4—H4	120.9	C9^i^—C9—H9B	109.0
N1—C5—C4	123.80 (12)	C8—C9—H9B	109.0
N1—C5—H5	118.1	H9A—C9—H9B	107.8
			
C6—N2—N3—C7	−0.50 (14)	C2—C3—C6—N2	−174.70 (13)
C5—N1—C1—C2	0.29 (19)	C4—C3—C6—N2	3.7 (2)
N1—C1—C2—C3	1.2 (2)	C2—C3—C6—O1	2.71 (17)
C1—C2—C3—C4	−1.37 (19)	C4—C3—C6—O1	−178.93 (11)
C1—C2—C3—C6	177.01 (11)	N2—N3—C7—O1	0.93 (14)
C2—C3—C4—C5	0.23 (18)	N2—N3—C7—S1	−177.95 (10)
C6—C3—C4—C5	−178.17 (11)	C6—O1—C7—N3	−0.96 (13)
C1—N1—C5—C4	−1.56 (19)	C6—O1—C7—S1	178.10 (8)
C3—C4—C5—N1	1.3 (2)	C8—S1—C7—N3	−0.56 (14)
N3—N2—C6—O1	−0.09 (14)	C8—S1—C7—O1	−179.42 (9)
N3—N2—C6—C3	177.42 (12)	C7—S1—C8—C9	177.46 (9)
C7—O1—C6—N2	0.61 (13)	S1—C8—C9—C9^i^	−69.35 (15)
C7—O1—C6—C3	−177.20 (11)		

Symmetry codes: (i) −*x*, −*y*+1, −*z*+1.

## References

[bb1] Al-Talib, M., Tashtoush, H. & Odeh, N. (1990). *Synth. Commun.* **20**, 1811–1817.

[bb2] Nakagawa, Y., Nishimura, K., Izumi, K., Kinoshita, K., Kimura, T. & Kurihara, N. (1996). *J. Pestic. Sci.* **21**, 195–201.

[bb3] Rigaku/MSC (2005). *CrystalClear* Molecular Structure Corporation, The Woodlands, Texas, USA, and Rigaku Corporation, Tokyo, Japan.

[bb4] Sheldrick, G. M. (2008). *Acta Cryst.* A**64**, 112–122. 10.1107/S0108767307043930 18156677

[bb5] Wang, H., Gao, Y. & Wang, W. (2010). *Acta Cryst.* E**66**, o3085. 10.1107/S1600536810044442 PMC3011391 21589392

[bb6] Wang, W., Gao, Y., Xiao, Z., Yao, H. & Zhang, J. (2011). *Acta Cryst.* E**67**, o269. 10.1107/S1600536810052979 PMC3051504 21522961

[bb7] Zhang, Z.-H., Li, C.-P., Tian, Y.-L. & Guo, Y.-M. (2007). *Acta Cryst.* E**63**, m3044.

[bb8] Zhao, B., Liu, Z., Gao, Y., Song, B. & Deng, Q. (2010). *Acta Cryst.* E**66**, o2814. 10.1107/S1600536810040729 PMC3009284 21589005

